# Development and psychometric testing of a clinical reasoning rubric based on the nursing process

**DOI:** 10.1186/s12909-023-04060-3

**Published:** 2023-02-07

**Authors:** Nargess Ramazanzadeh, Akram Ghahramanian, Vahid Zamanzadeh, Leila Valizadeh, Saeideh Ghaffarifar

**Affiliations:** 1 Students’ Research Committee, Nursing and Midwifery School, Tabriz University of Medical Sciences, Tabriz, Iran; 2 Department of Medical-Surgical Nursing, Faculty of Nursing and Midwifery, Tabriz University of Medical Sciences, Tabriz, Iran; 3 Department of Medical-Surgical Nursing, Nursing and Midwifery School, Shahid Beheshti University of Medical Sciences, Tehran, Iran; 4 Department of Pediatric Nursing, Nursing and Midwifery School, Shahid Beheshti University of Medical Sciences, Tehran, Iran; 5 Medical Education Research Center, Health Management and Safety Promotion Research Institute, Tabriz University of Medical Sciences, Tabriz, Iran

**Keywords:** Clinical reasoning, Education, Evaluation, Nursing process, Rubric

## Abstract

**Background:**

To facilitate the development of clinical reasoning skills in nursing students, educators must possess the ability to teach and evaluate them. This study aimed to describe the development and validation process of an analytic rubric of clinical reasoning skills based on the nursing process in undergraduate nursing students.

**Methods:**

A seven-step method was used for rubric development. The initial validation process of the rubric of clinical reasoning was performed with the participation of key stakeholders to assess its face and content validity as well as applicability in the classroom and bedside. An initial pilot test was performed based on scenario-based examinations in the nursing process training course so that convergent validity was used to show how closely the new scale is related to the previous measure for evaluating students’ tasks. Internal consistency and inter-rater correlation coefficient measurement for reliability were assessed.

**Results:**

The rubric to assess clinical reasoning skills was developed into eight categories according to the five stages of the nursing process. Content and face validity of the rubric were done qualitatively and resulted in a clear, simple rubric relevant to clinical reasoning skills assessment. The convergent validity was confirmed by the conventional method. The reliability was approved by a high inter-rater correlation coefficient based on the assessment by two random independent raters.

**Conclusion:**

The clinical reasoning meta-rubric developed in this study meets the purpose of the study. This analytical rubric can be applied to guide teaching and learning as well as evaluate clinical reasoning based on the findings. Testing the applicability confirmed its validity and reliability for assessing clinical reasoning skills in nursing process education during the undergraduate nursing program.

**Supplementary Information:**

The online version contains supplementary material available at 10.1186/s12909-023-04060-3.

## Background

As a tool to evaluate student work or assignments, a rubric is a coherent set of specific criteria describing the levels of performance quality [1]. A rubric is like a blueprint indicating mastery of skills or performance content [2]. In 2005, after a long-term effort to promote the value of liberal education, the Valid Assessment of Learning in Undergraduate Education (VALUE) project began to develop and publish rubrics [3]. This event highlighted the importance of rubrics in education.

In educational technology, a rubric refers to the standard of students’ performance [4], to evaluate their assignments [1, 2]. Although rubrics can be valid and reliable grading tools for instructors [5], they are more than merely guiding tools for grading papers, projects, and academic tests [1]. In addition to being an evaluation tool, they can be used to accurately describe the acceptance level of performance for each part of an assignment [5, 6], allowing coherent and unbiased evaluation [7]. Moreover, empowering students in independent learning requires involving them in the learning process and self-assessment. A rubric prepares students for the learning experience and facilitates evaluation [8], through the students’ and instructors’ perspectives and areas that need improvement [2]. Therefore, the continuous assessment of students’ learning and effective assessment of educational quality are also guaranteed [8]. Furthermore, the standard level determined in a rubric for the performance [2, 8] can motivate students for more detailed and accurate learning [8].

**Fig. 1:**
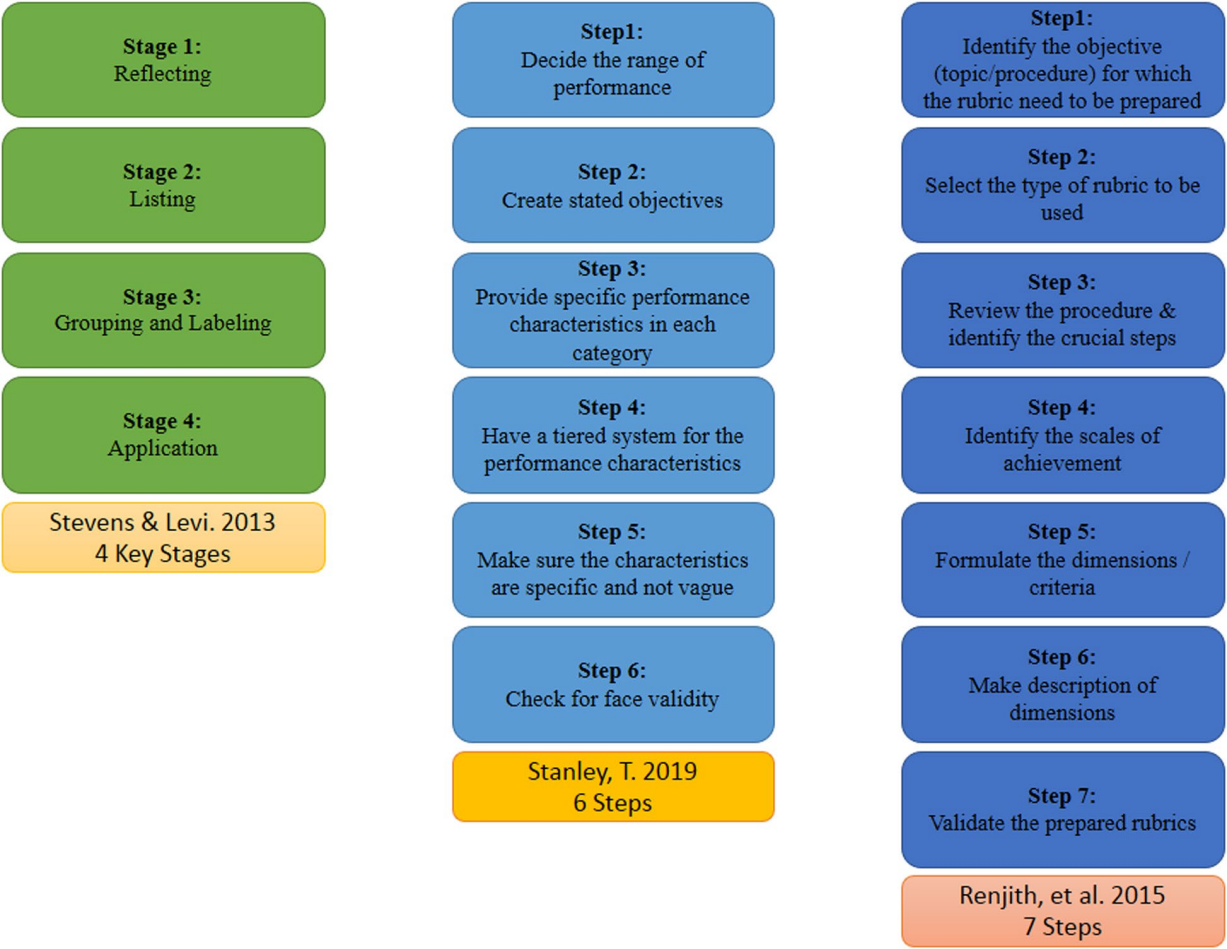
Various methods of rubric development and their step-by-step descriptions

Figure 1 shows different methods to design and develop various rubrics with different complexity levels [2, 4, 5]. In general, the final product of any rubric development method should contain its main constituents, including the title as a description of the performance being evaluated, the scale of achievement for quantitative or qualitative scoring, dimensions as the components of evaluation, and a description for each scale of dimensions as the performance levels [4].

Rubrics are categorized based on their functional methods [1, 2, 4] in terms of the considered domain and field of the task as the main aspects of rubrics differentiation [1, 2]. The main categories are divided by function or focus of a rubric [1, 2, 4]. In terms of singular or simultaneous functioning of items, rubrics are respectively categorized as analytical and holistic rubrics, while the generality or task-specificity of a rubric, represents how focused it is, so they are respectively named as generic/general and task-specific rubrics [1, 2, 4]. Furthermore, rubrics can be formative for monitoring the learning or summative for assessing cumulative/culminated learning [2]. Selecting the type of rubric depends on the task being evaluated, and there is no superior type to others [4].

Thinking ability and its process are essential and dedicated skills in the nursing profession [9]. Clinical reasoning (CR) is a crucial component of thinking about healthcare issues [6]. CR is defined as a skill, process, or outcome where nurses observe and collect data for the diagnosis and treatment of patients and make the best decision to resolve the problems accordingly [10]. Thus, it is essential to teach nursing students (NSs) thinking skills to achieve sufficient competency in providing patient care [9]. CR and critical thinking (CT) are core competencies of nursing practice [6, 11–13], which are in line with better outcomes of clinical judgments [6, 13]. Therefore, developing thinking skills is necessary to bridge theory and clinical practice to achieve more beneficial nursing practice [6, 11, 12]. Based on Facione’s study CT is a purposeful cognitive skill [13, 14] and commitment is the highest level of CT [15]. At this level, it is necessary to obtain specific competencies (diagnostic reasoning, clinical inference, and clinical decision-making) along with the general ones for nursing process (NP) competency [15]. Therefore, faculty members must develop methods to gain insight into students' CT to comprehend their learning and CR development [16]. At their best, based on the nature of the nursing profession, these evaluation methods must be applied both in simulated and actual clinical settings [10]. In addition to using methods such as problem-based learning [17] and simulation along with traditional lectures [11, 18], Alfaro-LeFevre introduced 17 facilitating integrated skills to promote CT and CR [6]. These CR skills are identifying assumptions, assessing systematically and comprehensively, checking accuracy and reliability as validating data, distinguishing normal from abnormal/identifying signs and symptoms, making inferences as drawing valid conclusions, clustering related cues or data, distinguishing relevant from irrelevant, recognizing inconsistencies, identifying patterns, identifying missing information, promoting health by identifying and managing risk factors, diagnosing actual and potential problems, setting priorities, determining patient-centered or client-centered outcomes, determining individualized interventions, evaluating and correcting thinking as self-regulation and determining a comprehensive plan/evaluating and updating the plan [6]. Likewise, self-assessment guarantees the awareness of performance, abilities, development in thinking, and performance in oneself [6]. Evaluating the effect of strategies or tools on the development of reasoning and CT in NS, Lasater, and Nielsen indicated that concept-based learning activities can deepen the thinking process as clinical learning strategies [19]. According to American Nurses Association standards, NP, as a fundamental concept in nursing, is a CT model for problem-solving and decision-making based on a holistic approach that includes all crucial actions conducted by nurses as assessment, diagnosis, outcome identification, planning, implementation and evaluation for providing quality individualized client care [20]. Also, NP is the basis for CR in clinical decision-making [6, 20, 21]. Therefore, to teach nurses to “think like a nurse”, it is necessary to teach clinical reasoning and judgment based on the NP model [6, 21].

Given that rubrics have become an undeniable part of nursing education and evaluation [4], weak and inconsistent rubrics in nursing education are causing challenges for the training, evaluating, and providing feedback to students on CR skills [22]. It is essential to consider that it will be necessary to develop evaluation methods incorporating all CR components to ensure obtaining CR competency [10]. Therefore, this study aimed to develop and conduct psychometric testing of an analytical rubric for CR education, learning, and evaluation based on NP.

## Methods

### Rubric development

**Fig. 2:**
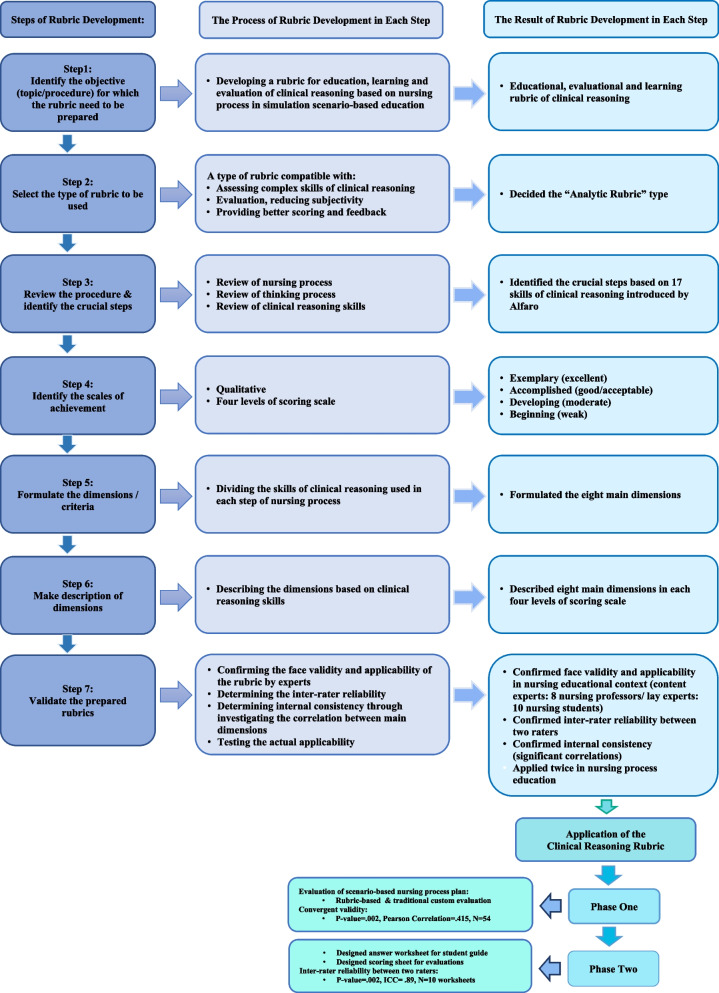
Stages of the Clinical Reasoning Rubric development and its utilization

In this methodological study, the seven-step rubric development method suggested by Renjith et al. was used to develop a CR education, learning, and evaluation rubric [4]. In addition to being an external evaluation tool based on instructors, it can be appropriate for students’ self-assessment and facilitate learning through feedback. In the first and second steps of rubric development, the purpose (CR education, learning, and evaluation based on NP) and type of the rubric (analytical) were determined. During the third step, the research team reviewed 17 CR skills introduced by Alfaro-LeFevre [6]. In the fourth step, the scoring scale was prepared for different levels of CR performance from beginning to exemplary [23, 24]. Each scoring level could acquire both quantitative and qualitative scores based on the general or specified description provided for the skill levels of each dimension in different situational applications. For example, if the rubric is supposed to be used for CR assessment in a scenario-based education, the assessor can specify the dimensional descriptions into scenario requirements according to the general available ones for more objective scoring. The conventional numeric scoring from 1 to 4 was used for better statistical analysis from weak to excellent in the current study. In the fifth step, eight main dimensions were extracted based on NP conceptual framework used for the CR rubric development. This framework organizes care as the main activity in nursing practice through five cyclic steps, i.e., assessment, diagnosis, planning, implementation, and evaluation of outcome state achievement [25, 26]. The eight dimensions included: 1) assessing systematically and comprehensively, 2) distinguishing normal from abnormal/identifying signs and symptoms, 3) clustering related cues (data), 4) diagnosing problem-focused, risk and health promotion problems/writing nursing diagnosis statement, 5) setting priorities, 6) determining patient/client-centered outcomes, 7) determining individualized nursing interventions, and 8) determining a comprehensive plan/evaluating and updating the plan. Then, the leading eight CR dimensions were accordingly divided into steps of NP, and based on experts’ opinions, qualitative content validity was confirmed. In the sixth step, each scoring level of dimensions was unequivocally and precisely described. Ultimately, psychometric testing was conducted for the developed rubric in the seventh step, which is explained in the following. The detailed steps of the rubric development method in the current study is shown in Fig. 2.

The COSMIN checklist (COnsensus-based Standards for selecting health status Measurement INstruments) [27, 28] for a well-developing instrument was utilized.

### The validity of the rubric

#### Initial validation process

In the initial validation process of the CR rubric, experts were selected from four groups of key stakeholders, including experts in nursing education and CR evaluation (*n* = 4), clinical instructors (*n* = 2), NP instructors (*n* = 2) as content experts, and undergraduate NSs except for freshmen (*n* = 10) as lay experts. In this study, both face and content validity evaluations were performed qualitatively. The lay experts were expected to think aloud about their understanding and how they could interact with the content of CR rubric through cognitive interviews [29] in order to optimize the clarity, comprehensibility, and quality of each dimensional phrase. The content experts were asked in a paper–pencil survey to provide their opinions, suggestions and explanation on the phrasing, structure and organization of the selected eight CR skills in the steps of NP and the description of students' performance levels in the developed rubric. In addition, the experts' panels were asked to comment on the importance of the CR rubric's dimensions, clarity, simplicity, and usability. The survey included the following questions: 1) Do the constituting parts of the rubric seem essential and appropriate for the CR rubric based on NP? 2) Which parts of the rubric are hard to use? 3) What are your suggestions to improve the rubric? 4) Are eight dimensions with four scoring levels and descriptions of each level sufficient? After applying their suggested corrections and designing an answer-worksheet for recording students’ assignments, the rubric was given to 10 NSs, and they were asked to examine the different parts of the rubric as mentioned and express their opinions. The opinions of this group were used to revise the phrasing, structure, scoring, and description of each performance level.

Then, two faculty members used the developed rubric and designed worksheet to evaluate students’ assignments during the NP education in the “Basic Nursing Concepts” course. They utilized the rubric to evaluate students’ assignments concerning writing a nursing care plan based on NP and then provided their feedback on the applicability of the rubric and usability of the worksheet in recording students’ assignments as a facilitator to better understand the structure and organize the answers.

#### Convergent validity of the rubric

During the COVID-19 pandemic, the theoretical courses in the nursing schools of Iran were held online on the educational application of Tabriz University of Medical Sciences, known as NAVID. From February to June 2020, the second semester of the educational calendar in the Iranian academic year, the rubric was utilized in NP education to evaluate the uploaded assignments of second-semester NSs.

A study was designed during the mentioned period to examine the convergent validity of the developed CR rubric. After theoretical education of the NP to second-semester NSs (*n* = 55), the research team asked for an assignment. The students were expected to write a palliative nursing care plan for a metastatic cancer case based on NP. The two course instructors scored the students’ assignments separately, one used the developed rubric, and the other used the conventional method. In absence of grading rubrics, the conventional method is used as a routine evaluation process for written assignments. Therefore, the content of each assignment was judged in terms of the quantity and quality of the answers, based on the instructor’s expectation and lesson plan goals as the conventional method of evaluation. The correlation between these two scores was analyzed using Pearson’s correlation coefficient at *p*-value < 0.05 in SPSS 24.

#### Applicability and effectiveness of the rubric worksheet in scenario-based assignments

From September 2020 to January 2021, the first semester of the educational calendar in the Iranian academic year, the rubric was reutilized to evaluate the scenario-based assignment of second-semester NSs (*n* = 50) during the NP education, recorded on developed answer-worksheets. They were asked to analyze a laparoscopic appendectomy case, select two high-priority nursing diagnoses, and record a nursing care plan on the answer-worksheet based on CR rubric structure. The students’ assignments were evaluated only by the final developed rubric and the scoring sheet prepared for this purpose. The students’ scores in this semester were compared with those of students in the previous semester to examine the facilitating role of the worksheet in the scenario-based assignment. Moreover, the course instructors and students were interviewed concerning the effectiveness of utilizing the worksheet to record the care plan by students and evaluate their answers by instructors.

### The reliability of the rubric

During the NP education for second-semester NSs from September 2020 to January 2021, the scenario-based assignment for a case of laparoscopic appendectomy, ten assignments were selected randomly and scored by the two instructors using the newly developed rubrics. They were trained how to use the rubric; they also used an agreed-upon adapted rubric fitting to the determined scenario as a guide (Supplementary file 1). The inter-rater reliability was assessed calculating the inter-rater intra-class correlation coefficient (ICC) measurement using Pearson correlation coefficient for a two-way random absolute agreement ICC. Moreover, the correlation between main dimensions (each one contains a single phrase) and total CR rubric mark was investigated as dimension-to-dimension and dimension-to-total correlation to ensure its internal consistency.

## Results

### Validity results

#### Face validity and applicability of the rubric

The faculty members indicated that the eight dimensions of CR skills are comprehensive and sufficient and can fit into five steps of the NP. Expert panel members agreed that these categories would help identify the specific components of CR.

Following this step, the developed rubric was used in a sample of second-semester NSs in a course on “Basic Nursing Concepts” to evaluate scenario-based assignments. The instructors also reflected on their experience in utilizing the rubric compared to their previous method.

The instructors of this course, who used CR rubric to grade and evaluate students' assignments, found it more valuable and accurate but believed that it was more time-consuming than the method they previously used. They suggested that designing an answer-worksheet based on the developed rubric be more helpful for the students to understand and accomplish the expected requirements of the course in their assignment records in a single and similar structure for better comparison and Judgement. Also, they suggested that preparing a scoring sheet for instructors can facilitate the students’ evaluation and make it more objective. These comments led us, to review the rubric and prepare the answer-worksheet and scoring sheet (Supplementary files 2 and 3).

In the cognitive interview with the students, the rubric and these two newly designed sheets were given to 10 students who had previously participated in the course mentioned above, and they approved them. They practiced recording a scenario on an answer-worksheet and found it easy and comprehensible to use. The optimizing process of the developed rubric followed qualitative comments of content and lay experts who reviewed and utilized the rubric initially and after its revisions, which resulted in designing students' worksheet and the assessors' scoring sheet as well as the final developed CR rubric.

Eventually, the final rubric was confirmed after face validity and applicability in the group of content experts and students (Table 1).

**Table 1:** The rubric of Clinical Reasoning based on the nursing process

Nursing Process Steps	Dimensions	Scale and Description			
		Excellent (Exemplary)	Good (Acceptable/Fair)	Average (Moderate/Developing)	Weak (Beginning)
Assessment	Assessing Systematically and Comprehensively	Selects proper initial or focused assessment form as needed, considering various sources of data; and performs a comprehensive and purposeful nursing and medical assessment of client	Selects proper initial or focused assessment form as needed, considering less various sources of data; performs a fairly comprehensive and purposeful nursing and medical assessment of client, may miss some subtle data	Misses to select proper initial or focused assessment form as needed, without considering various sources of data; performs an incomplete and unstructured nursing or medical assessment of client, misses some important data	Misses to select proper initial or focused assessment form as needed, without considering various sources of data; confronts problem in nursing or medical assessment of client
	Distinguishing Normal from Abnormal/Identifying Signs and Symptoms	Analyzes all client data, correctly decides which is in the normal or abnormal range, and correctly recognizes the signs and symptoms of health problems	Analyzes most of the important client data, correctly decides which is in the normal or abnormal range, and correctly recognizes most of the signs and symptoms of health problems	Analyzes some of the important client data, fairly decides which is in the normal or abnormal range, and fairly recognizes some of the signs and symptoms of health problems	Confronts problem in analyzing client data, deciding the normality range, and recognizing the signs and symptoms of health problems
Nursing Diagnosis	Clustering Related Cues (Data)	Identifies all relevant data in each domain, then classifies relevant data of various domains into meaningful clusters for each health condition, identifying the relationships and existing patterns among the data and risk factors	Identifies most of the important relevant data in each domain, then classifies most of the relevant data of various domains into meaningful clusters for each health condition, fairly identifying the relationships and existing patterns among the data and risk factors; may miss some subtle data	Identifies some of the relevant data in each domain, then classifies some of the relevant data of various domains into meaningful clusters for each health condition, to some extent identifying the relationships and existing patterns among the data and risk factors; misses some important data	Confronts problem in identifying relevant data in each domain, and classifying relevant data of various domains into meaningful clusters for each health condition; there are flaws and problems in identifying the relationships and existing patterns among the data and risk factors
	Diagnosing Problem-focused, Risk and Health Promotion Problems/Writing Nursing Diagnosis Statement	Identifies all problem-focused, risk and health promotion nursing diagnoses completely based on NANDA-I nursing diagnoses, and writes the diagnosis statement exactly according to the PES template	Identifies most of the problem-focused, risk and health promotion nursing diagnoses fairly based on NANDA-I nursing diagnoses, and writes the diagnosis statement fairly according to the PES template	Identifies some of the problem-focused, risk and health promotion nursing diagnoses to some extent based on NANDA-I nursing diagnoses, and writes the diagnosis statement to some extent according to the PES template	Confronts problem in identifying the problem-focused, risk and health promotion nursing diagnoses based on NANDA-I nursing diagnoses, and writing the diagnosis statement according to the PES template
Planning	Setting Priorities	Prioritizes the client's list of nursing diagnoses completely based on the most important and priority life-threatening problems (ABCDES) or patient needs (according to the Maslow's needs pyramid) and patient preferences	Prioritizes the client's list of nursing diagnoses fairly based on the most important and priority life-threatening problems (ABCDES) or patient needs (according to the Maslow's needs pyramid) and patient preferences	Prioritizes the client's list of nursing diagnoses to some extent based on the most important and priority life-threatening problems (ABCDES) or patient needs (according to the Maslow's needs pyramid) and patient preferences	Confronts problem in prioritizes the client's list of nursing diagnoses based on the most important and priority life-threatening problems (ABCDES) or patient needs (according to the Maslow's needs pyramid) and patient preferences
	Determining Patient/Client-Centered Outcomes	Selects the appropriate expected outcome(s) (based on NOC) correctly, and sets the objectives complete and according to the SMART template	Selects the appropriate expected outcome(s) (based on NOC) fairly correct, and sets the objectives fairly complete and according to the SMART template	Selects the appropriate expected outcome(s) (based on NOC) to some extent correct, and sets the objectives incompletely and less according to the SMART template	Confronts problem in selecting the appropriate expected outcome(s) (based on NOC), and setting the objectives according to the SMART template
	Determining Individualized Nursing Interventions	Selects all independent (based on NIC and evidence), dependent and collaborative nursing interventions and activities considering the relevant/risk factors or defining characteristics of diagnosis; and involves stakeholders and caregivers in interventions adjustment	Selects most of the independent (based on NIC and evidence), dependent and collaborative nursing interventions and activities fairly considering the relevant/risk factors or defining characteristics of diagnosis; and fairly involves stakeholders and caregivers in interventions adjustment	Selects some of the independent (based on NIC and evidence), dependent and collaborative nursing interventions and activities to some extent considering the relevant/risk factors or defining characteristics of diagnosis; and less involves stakeholders and caregivers in interventions adjustment	Confronts problem in selecting the independent (based on NIC and evidence), dependent and collaborative nursing interventions and activities considering the relevant/risk factors or defining characteristics of diagnosis; and involving stakeholders and caregivers in interventions adjustment
Evaluation	Determining a Comprehensive Plan/Evaluating and Updating the Plan	Evaluates the effectiveness of the care plan and the client's progress toward expected outcomes continually and ongoing and based on the changes in client's condition; and makes the necessary changes at each step of the care plan, and updates it	Fairly evaluates the effectiveness of the care plan and the client's progress toward expected outcomes continually and ongoing and based on the changes in client's condition; and mostly makes the necessary changes at each step of the care plan, and updates it	Evaluates the effectiveness of the care plan and the client's progress toward expected outcomes to some extent continually and ongoing and based on the changes in client's condition; and sometimes makes the necessary changes at each step of the care plan, and updates it	Confronts problem in continual and ongoing evaluation of the effectiveness of the care plan and the client's progress toward expected outcomes based on the changes in client's condition; and making the necessary changes at each step of the care plan, and updating it

*NANDA-I* North American Nursing Diagnosis Association-International, *PES* Problem—Etiology—Signs & Symptoms, *ABCDES* Airway—Breathing—Circulation—Disabilities – Exposure- Stimuli, *NOC* Nursing Outcomes Classification, *SMART* Specific—Measurable—Attainable/Accessible—Real – Timing, *NIC* Nursing Interventions Classification

### Results of convergent validity

The evaluation of the scenario-based assignment of 55 s-semester undergraduate NSs (31 females and 24 males with an average age of 21.03 ± 1.46) by two independent raters showed a significant moderate correlation between the scores of the conventional method and CR rubric-based evaluation (*p* = 0.002 & *r* = 0.415) (Table 2).

**Table 2:** Results for convergent validity

Evaluation methods	Mean	Standard Deviation	Possible Range Score	Results of correlation r (*p-value*)
Conventional	4.34	0.79	0–5	0.415 (0.002)
Rubric	12.67	2.05	8–32	

### Effectiveness of the worksheet to record care plan in scenario-based assignments

Fifty second-semester NSs (25 females and 25 males with an average age of 20.91 ± 1.73) recorded their assignments on the answer-worksheet in this study stage. The comparison of the students’ during the two semesters in terms of using the worksheet, demonstrated a significant increase in the mean scores in three dimensions, namely assessing systematically and comprehensively (*p* < 0.001), distinguishing normal from abnormal/identifying signs and symptoms (*p* = 0.001), and determining individualized nursing interventions (*p* = 0.046). There was no significant difference between the two groups of students in the overall rubric score and other dimensions, including clustering related cues (data); diagnosing problem-focused, risk and health promotion problems/writing nursing diagnosis statements; setting priorities; determining patient/client-centered outcomes; and determining a comprehensive plan/evaluation and updating the plan (Table 3). In their cognitive interviews, the students' feedback indicated that they found the worksheet helpful and student-friendly in understanding what was asked and where to write the answers. The instructors who utilized the CR rubric to evaluate the students’ work found the evaluation process easier and less time-consuming.

**Table 3:** Comparison of the mean scores of students in two academic semesters in terms of using the worksheet

Group		Rubric and dimensions scores Mean (SD)								
	Nursing process course time	Rubric Mark	Assessment	Normal Abnormal Signs & Symptoms	Clustering Data	Nursing Diagnosis PES	Prioritizing	Determining Outcomes	Determining Interventions	Evaluation
Group A (Answer the task without a worksheet) *N* = 55	February to June 2020	12.67 (2.05)	1.91 (0.55)	1.82 (0.38)	1.24 (0.47)	1.82 (0.51)	1.42 (0.49)	1.60 (0.49)	1.85 (0.52)	1.02 (0.13)
Group B (Answer the task on the worksheet) *N* = 50	September 2020 to January 2021	13.38 (1.81)	2.36 (0.59)	2.00 (0.00)	1.22 (0.5)	1.66 (0.55)	1.36 (0.52)	1.68 (0.47)	2.04 (0.4)	1.02 (0.14)
*p-value*		0.066	< 0.001	.001	.864	.133	.561	.399	.046	.946
CI:										
Lower		-1.461	-.674	-.291	-.173	-.049	-.140	-.267	-.368	-.055
Upper		.047	-.228	-.073	.205	.365	.256	.107	-.003	.052

*SD* Standard Deviation, *CI* Confidence Interval

### Results of the reliability of the rubric

The correlation between eight rubric dimensions (each dimension contains 1 phrase) and total rubric mark indicated that most of them are correlated at 0.01 level, and internal consistency exists (Table 4). The analysis of the correlation between the scores of two instructors, who separately evaluated the worksheets of ten students for a scenario-based assignment, showed an appropriate correlation at 95% confidence intervals using a two-way random absolute agreement inter-rater intra-class correlation coefficient with a Pearson correlation coefficient of 0.89, approving the good reliability of the developed rubric (Table 5).

**Table 4:** Correlation between main dimensions in clinical reasoning rubric

Dimensions	Assessment	Normal/Abnormal Signs/Symptoms	Clustering Data	Nursing Diagnosis	Prioritizing	Determining Outcomes	Determining Interventions	Evaluation
Total Rubric Mark	.594^a^	.399^a^	.461^a^	.684^a^	.687^a^	.530^a^	.554^a^	.106
Assessment	1	.436^a^	.065	.330^a^	.182	.055	.155	.199^b^
Normal/Abnormal Signs/Symptoms		1	-.115	.269^a^	.122	.161	-.039	.045
Clustering Data			1	.154	.297^a^	.151	.265^a^	-.066
Nursing Diagnosis				1	.511^a^	.194^b^	.205^b^	.067
Prioritizing					1	.306^a^	.251^a^	.030
Determining Outcomes							.327^a^	-.185
Determining Interventions							1	.017
Evaluation								1

^a^Correlation is significant at the 0.01 level (2-tailed)

^b^Correlation is significant at the 0.05 level (2-tailed)

**Table 5:** Comparison of the mean scores of students by two raters and inter-rater reliability using clinical reasoning rubric

Rater	Mean	Std. Deviation	Possible Range Score	Inter-rater Intra-Class Correlation Coefficients (Inter-rater ICC) *(p*-value)
Rater 1	19.00	3.651	8–29	0.89 (.002) Average Measures (Two-way random absolute agreement)
Rater 2	19.30	5.012	8–29	

## Discussion

The current study developed a rubric of CR training and evaluation in eight dimensions. The results confirm the fitness of these eight dimensions with the steps of the NP. An analytical rubric objectively evaluates complex skills and their scoring by describing different dimensions of performance [2, 4]. Focusing on dimensions of CR as a complex thinking skill was one of the strengths of this study in developing an analytic rubric. Also, practical revisions were made based on discussions and assessments of experts to make it more comprehensible. According to the available literature, a developed rubric must have good validity [2, 4, 30], and the current CR rubric indicated appropriate face validity and applicability from the view of different content and lay experts. Given that the selected experts, including experts in nursing education, instructors of NP, clinical instructors, and students, were critical stakeholders in utilizing the CR rubric, their confirmation of face validity indicated the validity of this educational tool in terms of factors such as the relationship of dimensions and the scaling, ease of answering and evaluating, clarity of descriptions, and the judgmental items [31]. The convergent validity of the rubric (*r* = 0.415) showed a moderate correlation [32] between the rubric-based evaluation and the conventional evaluation method. However, positive correlation indicated more convergent validity [33]; it should be considered that the conventional evaluation is less objective and details might be lost, or the student's excessive explanation may obscure the accuracy of the responses. Therefore, a moderate positive correlation seems acceptable and appropriate for the developed rubric. It allows the rater to evaluate in a less biased manner and more objectively, helping focus on the necessary details rather than the number of provided explanations. Thus, moderate convergent correlation can demonstrate the validity of the developed rubric. Accordingly, this analytical rubric can help develop thinking skills, specifically CR, which unfortunately is mistakenly considered equal with other thinking skills or evaluated under different topics.

According to Lunsford’s thesis in 2020, analytical grading rubrics create stability in evaluation, identifying competent students and increasing students’ and instructors’ self-efficacy [34]. The rubric developed in this study can also help identify students with exemplary CR skills and increase self-efficacy in hard-working students and instructors who correctly evaluate and identify them. Furthermore, it should be noted that the CR rubric constructed by researchers in a study by Kim and Kim was developed to create a consistent scoring construct [18]. The main limitations of their rubric are the absence of all dimensions of CR skill and different scoring among the dimensions. In contrast, in the rubric developed in this study, details of CR skills are covered comprehensively, which allows evaluation of this complex thinking skill in NP education with the ability of efficient quantitative and qualitative scoring. Additionally, the rubric developed by Furze et al. (2015) aimed to evaluate CR in physical therapy students and provided observation of CR level with accurate descriptions [16]. Congruently, the rubric developed in this study to evaluate CR in NSs provides accurate descriptions of skill levels in each dimension and applies in various educational situations, introducing it as a “meta-rubric”.

Considering the practical applicability of this analytical rubric in simulated scenario-based education and actual clinical situations, providing proper feedback to students is crucial. In addition, simulation-based NP education helps develop communication skills, self-confidence, and understanding of the NP; it can provide an interactive learning experience [35]. In fact, one of the characteristics of analytical rubrics is providing more comprehensive feedback to students [2, 4]. Czajka et al. (2021) introduced that providing detailed feedback to students is a resolved limitation in analytical rubrics [36]. Similarly, with the improvement of descriptions in each dimension of the rubric according to the applied performance standard in each level, these limitations were resolved in this study to a great extent.

Given the intended educational setting of the instructor/rater, the developed CR rubric enhances the capability of providing efficient feedback to NSs along with self-assessment. Moreover, it should be noted that given the applicability of the developed rubric in scenario-based assignments and practices in simulated and actual clinical education, the instructor/rater should determine the standard level of each dimension according to the selected scenario or the actual client and use it as the educational, learning, and evaluation blueprint. This can be carried out by preparing an adapted rubric that fits the scenario or the educational situation and increases objectivity and inter-rater agreement when more than one rater is required. As expressed by Lunsford (2020), a rubric utilized for scoring should be stable and have a high level of inter-rater reliability [34]. In examining the reliability of the developed rubric, using the adapted rubric fitting to the determined scenario, the inter-rater agreement was 89%, which is acceptable and appropriate and indicates its proper reliability [37, 38]. Moreover, the significant correlation between dimensions of newly developed rubric confirmed the internal consistency as a reliability criterion.

Considering that NP education is offered within a course called “Basic Nursing Concepts” in the second-semester of the undergraduate nursing program in Iran, the developed rubric was utilized in the first level of NP education in both applied phases to evaluate the second-semester NSs, and it showed a lower-than-average level of CR skills. This finding can be explained by the results of Leijser and Spek (2020), as in their study, the intermediate NSs were successful in CR levels in an educational environment, which highlighted the level of education and amount of clinical healthcare internship [39]. Moreover, Kim and Kim (2015), who used their researchers-constructed rubric to evaluate CR in third-year students, reported higher-than-average CR scores [18], indicating students’ clinical care experience. Therefore, considering the target group for the application and assessment of the developed rubric that included two groups of the second-semester NSs, the lower-than-average level of CR in the participating students can be rationalized by their limited clinical care experience and clinical interaction with patients and clients.

NP education should continue throughout the undergraduate nursing program, and the level of thinking skills achievement should increase with every passing semester. However, this can be one of the current study’s limitations as the target population was confined to the second-semester NSs according to the nursing curriculum in Iran. In nursing education, it is crucial to focus on education and conduct NP, as well as develop thinking skills, which requires the collaboration of the nursing education team to use beneficial educational methods.

### Implication for practice

The CR meta-rubric facilitates learning and assessing CR in undergraduate nursing education from freshman to internship in fundamental NP education. Moreover, the capability of self-assessment empowers the students to develop more appropriate thinking skills on their own. Therefore, this tool allows the stakeholders to monitor the progression of CR skills and reinforce it by providing timely and appropriate feedback. Furthermore, because it can be applied to various educational situations in virtual and actual situations, its significance is even more highlighted. In actual clinical settings, the rubric can also be effectively used to develop standard care plans, and evaluate students' performance.

## Conclusion

Thinking skills require standard evaluation to highlight their different dimensions in students’ education and learning given their complex nature. In addition to creating an educational and learning environment based on thinking skills, the developed meta-rubric, which focuses on CR based on NP, meets the purpose of the study. This analytical rubric can be applied to guide teaching and learning as well as evaluate CR based on the findings. In addition, it is applicable for assessing clinical reasoning skills in NP education during the undergraduate nursing program. Utilizing the CR rubric makes instructors and students more familiar with CR development by determining target educational, learning, and evaluation standards for case-based scenarios or actual, clinical and simulation situations where a client story exists.

## Supplementary Information

**Additional file 1.** Adapted rubric of Clinical Reasoning for laparoscopic surgery scenario.

**Additional file 2. **Worksheet for Clinical Reasoning Rubric.

**Additional file 3. **Scoring sheet for Clinical Reasoning Rubric.

## Data Availability

The data supporting this study's findings are available from the first author upon reasonable request.

## Abbreviations

COSMIN: COnsensus-based Standards for selecting health status Measurement INstruments
CR: Clinical Reasoning
CT: Critical Thinking
NP: Nursing Process
NS: Nursing Student
VALUE: Valid Assessment of Learning in Undergraduate Education
