# Supplementary material for: Development and psychometric testing of a clinical reasoning rubric based on the nursing process
Source: BMC Med Educ. 2023 Feb 7;23:98. doi: 10.1186/s12909-023-04060-3 (PMC9904873; doi:10.1186/s12909-023-04060-3)
Supplement: Supplementary file 1 — Additional file 1. Adapted rubric of Clinical Reasoning for laparoscopic surgery scenario. [file 12909_2023_4060_MOESM1_ESM.docx]

Supplementary file 1-Adapted Rubric of CR for Laparoscopic Surgery Scenario

The client Ms. KR was transferred to the surgical ward half an hour ago after a laparoscopic appendectomy. Assessment findings disclosed: HR = 102, frowning, abdomen tenderness while touching, three perforation wounds in the abdomen related to inserting endoscope and surgical instruments into the peritoneal cavity which were covered with three transparent sterile dressings. Also the pain severity was report 8 based on a numerical 10-degree scale.

Care plan is expected for two higher priority nursing diagnoses.

| Nursing Process Steps | Dimensions | Scale and Description | | | |
| --- | --- | --- | --- | --- | --- |
|  |  | Excellent  (Exemplary)  4 | Good  (Acceptable/Fair)  3 | Average  (Moderate/Developing)  2 | Weak  (Beginning)  1 |
| Assessment | Assessing Systematically and Comprehensively | Indicated all 6 items of data (either in the first box or in other parts of the worksheet).  Not mentioning the "three clear sterile dressings", can be ignored because the deviation from the normal state indicators are requested in the worksheet. | Indicated at least 4 items of data (either in the first box or in other parts of the worksheet).  Missing partial data includes:  "HR = 102, frowning" | Did not indicate at least one of the important data.  Important data includes:  "Pain intensity report, abdomen tenderness while touching, three perforation wounds of endoscope" | Identified less than 2 data or provided a description without indicating the data. |
|  | Distinguishing Normal from Abnormal/Identifying Signs and Symptoms | Distinguished all abnormal data, either in the first box or in other parts of the worksheet, especially in data clustering.  Identified all objective and subjective data correctly. | In addition to distinguishing the data in the first box, included most of the data in the data cluster.  Did not indicate the interpretation of high rate contrary to adding HR in client data or did not include the HR in clusters.  Misidentified at least one item of objective or subjective data. | In addition to distinguishing the data in the first box, included some of the data in the data cluster.  Misidentified over two items of objective or subjective data. | Did not include the Identified data of the first box into the clusters and did not indicate the deviation from the normal range for numeric data.  Misidentified the objective and subjective data. |
| Nursing Diagnosis | Clustering Related Cues (Data) | Determined at least two significant clusters of related data.  Completely acceptable clusters include related data for following Nursing Diagnoses:  Pain, Risk for infection and Impaired tissue integrity | Determined two significant clusters of most related data.  Acceptable clusters with incomplete data and including direct and indirect related data for following Nursing Diagnoses:  Pain, Risk for infection and Impaired tissue integrity | Determined at least one significant cluster with at least one related data of acceptable clusters for this scenario.  Acceptable clusters with incomplete data for following Nursing Diagnoses:  Pain, Risk for infection and Impaired tissue integrity  Indicated clusters with incomplete data for Nursing Diagnoses which are not related. | Did not cluster data or included data which are not related. |
|  | Diagnosing Problem-focused, Risk and Health Promotion Problems/Writing Nursing Diagnosis Statement | Recorded at least two of important diagnoses based on NANDA and the PES template.  The diagnosis of pain must be written correctly.  Fully accepted diagnoses:  Pain (acute), Risk for infection and Impaired tissue integrity | Recorded two acceptable and correct diagnoses based on NANDA with a slight defect in the name or the PES template.  One of the diagnoses must be Pain, even if confronted difficulty in diagnosing the type of pain.  Incorrectly used the word “Probability” instead of “Risk”.  If recorded one completely correct and one incomplete diagnosis. | Recorded one acceptable and correct diagnoses based on NANDA with a slight defect in the name or the PES template.  If the second diagnosis was incorrect, even if it was based on NANDA and the PES template or if one incomplete diagnosis with problematic writing.  Only wrote the name of nursing diagnosis (NANDA label). | Did not record any diagnosis or incorrect clinical judgment and problematic and non-priority diagnosis with incorrect writing and non-NANDA label. |
| Planning | Setting Priorities | Correctly prioritized at least two of the correct diagnoses.  Must have indicated the pain.  Order of completely acceptable diagnoses:  Pain (acute), Risk for infection and Impaired tissue integrity | Prioritized two of the correct diagnoses respectively.  Did not indicate the Pain but two other diagnoses were correct.  Confronted difficulty in prioritizing Pain but the other diagnosis was one of the correct diagnoses. | Indicated of the correct diagnoses as first priority.  Indicated the Pain without prioritizing and the other diagnosis was not correct. | Did not prioritize or indicated wrong diagnoses. |
|  | Determining Patient/Client-Centered Outcomes | Indicated at least two general outcomes that are correct and almost identical to the NOC (for example, pain relief instead of pain intensity or pain control) and set goals (at least one correct goal for each outcome) in SMART template (with at least 4 components, especially timing and type of measure). | Indicated at least two general and quantitative NOC outcomes, one of which is correct (according to the concept; for example, pain monitoring) and the goals (at least one correct goal) in SMART template (with at least 3 components). | Indicated at least one general outcome or a goal out of in priority items or near correct (perhaps different from the main concept; for example, attention to pain). | Did not record outcome or goal; wrong record of outcome or goal, recording intervention instead of outcome or goal. |
|  | Determining Individualized Nursing Interventions | Indicated at least two nursing interventions and important independent nursing activities (preferably based on NIC and evidence), and dependent and collaborative actions for each diagnosis. | Indicated at least one nursing intervention and important independent nursing activity (without attention to NIC but based on evidence or literature) for each diagnosis, and did not indicate dependent and collaborative actions. | Only indicated dependent and collaborative actions (related or not related) or medical orders.  Indicated at least one nursing intervention and independent nursing activity (based on evidence or literature). | Confronted problem in writing nursing interventions and independent activities, dependent and collaborative actions; or did not record. |
| Evaluation | Determining a Comprehensive Plan/Evaluating and Updating the Plan |  |  |  | This Item was not asked in the worksheet so all participants will get the same mark=1. |
