# Supplementary material for: Development and psychometric testing of a clinical reasoning rubric based on the nursing process
Source: BMC Med Educ. 2023 Feb 7;23:98. doi: 10.1186/s12909-023-04060-3 (PMC9904873; doi:10.1186/s12909-023-04060-3)
Supplement: Supplementary file 2 — Additional file 2. Worksheet for Clinical Reasoning Rubric. [file 12909_2023_4060_MOESM2_ESM.docx]

Supplementary file 2-Worksheet for Clinical Reasoning

| Dimensions | Student Records | |
| --- | --- | --- |
| Assessment | Identify the Objective Data and indicate if they are normal and abnormal: | Identify the Subjective Data and indicate if they are normal and abnormal: |
| Nursing Diagnosis | Cluster(s) of related data: | Related Nursing Diagnosis(es) of related cluster(s): |
|  | Write the Nursing Diagnosis statement(s) based on PES template: | |
| Planning | Prioritize and arrange the identified problems: | |
|  | Based on the identified problems, determine the expected outcomes of the client/patient (based on NOC) and set goals (based on SMART template). | |
|  | Based on the nursing diagnosis and expected outcomes of the client/patient, choose and record the appropriate interventions (independent, dependent, and collaborative) (based on NIC or evidence). | |
| Evaluation | Evaluate your care plan continually and ongoing; and considering the client's progress toward expected outcomes, make the necessary changes at each stage of the care plan and update it, as needed. | |
