# Supplementary material for: Development and psychometric testing of a clinical reasoning rubric based on the nursing process
Source: BMC Med Educ. 2023 Feb 7;23:98. doi: 10.1186/s12909-023-04060-3 (PMC9904873; doi:10.1186/s12909-023-04060-3)
Supplement: Supplementary file 3 — Additional file 3. Scoring sheet for Clinical Reasoning Rubric. [file 12909_2023_4060_MOESM3_ESM.docx]

| Supplementary file 3-Scoring Sheet for Clinical Reasoning Rubric  Student Name: The Observation Date/Time: Scenario No: | | | | | |
| --- | --- | --- | --- | --- | --- |
| Clinical Reasoning | Excellent  (Exemplary) | Good  (Acceptable/Fair) | Average  (Moderate/Developing) | Weak  (Beginning) | Observation Notes: |
| Assessment includes:   - Assessing Systematically and Comprehensively - Distinguishing Normal from Abnormal/Identifying Signs and Symptoms |  |  |  |  |  |
| Nursing Diagnosis includes:   - Clustering Related Cues (Data) - Diagnosing Problem-focused, Risk and Health Promotion Problems/Writing Nursing Diagnosis Statement |  |  |  |  |  |
| Planning includes:   - Setting Priorities - Determining Patient/Client-Centered Outcomes - Determining Individualized Nursing Interventions |  |  |  |  |  |
| Evaluation includes:   - Determining a Comprehensive Plan/Evaluating and Updating the Plan |  |  |  |  |  |
| Summary Comments: |  |  |  |  |  |
